# Optimal Versus Realized Trajectories of Physiological Dysregulation in Aging and Their Relation to Sex-Specific Mortality Risk

**DOI:** 10.3389/fpubh.2016.00003

**Published:** 2016-01-25

**Authors:** Konstantin G. Arbeev, Alan A. Cohen, Liubov S. Arbeeva, Emmanuel Milot, Eric Stallard, Alexander M. Kulminski, Igor Akushevich, Svetlana V. Ukraintseva, Kaare Christensen, Anatoliy I. Yashin

**Affiliations:** ^1^Biodemography of Aging Research Unit (BARU), Social Science Research Institute, Duke University, Durham, NC, USA; ^2^Groupe de Recherche PRIMUS, Department of Family Medicine, CHUS-Fleurimont, University of Sherbrooke, Sherbrooke, QC, Canada; ^3^The Danish Aging Research Center, University of Southern Denmark, Odense, Denmark; ^4^Department of Clinical Genetics, Odense University Hospital, Odense, Denmark; ^5^Department of Clinical Biochemistry and Pharmacology, Odense University Hospital, Odense, Denmark

**Keywords:** physiological dysregulation, stochastic process model, Mahalanobis distance, longitudinal data, mortality, sex differences

## Abstract

While longitudinal changes in biomarker levels and their impact on health have been characterized for individual markers, little is known about how overall marker profiles may change during aging and affect mortality risk. We implemented the recently developed measure of physiological dysregulation based on the statistical distance of biomarker profiles in the framework of the stochastic process model of aging, using data on blood pressure, heart rate, cholesterol, glucose, hematocrit, body mass index, and mortality in the Framingham original cohort. This allowed us to evaluate how physiological dysregulation is related to different aging-related characteristics such as decline in stress resistance and adaptive capacity (which typically are not observed in the data and thus can be analyzed only indirectly), and, ultimately, to estimate how such dynamic relationships increase mortality risk with age. We found that physiological dysregulation increases with age; that increased dysregulation is associated with increased mortality, and increasingly so with age; and that, in most but not all cases, there is a decreasing ability to return quickly to baseline physiological state with age. We also revealed substantial sex differences in these processes, with women becoming dysregulated more quickly but with men showing a much greater sensitivity to dysregulation in terms of mortality risk.

## Introduction

The aging process involves many physiological changes affecting the homeostatic state of the organism (1). Studies of biomarkers of aging thus have the potential to simultaneously shed light on the underlying aging process and to provide measurement tools for understanding how aging proceeds within an individual. While a large number of studies have examined changes in biomarkers during the aging process, almost all of these have considered markers one at a time and without reference to how optimal levels of the markers change with age [see, e.g., Ref. (2)]. However, it is clear on the one hand that optimal levels can and do change with age (3, 4), and on the other that information contained in biomarkers is highly redundant, non-linear, and complex (5–7). While some studies have succeeded in incorporating one or the other of these complexities, none have yet attempted both simultaneously. The need for new methodological developments that would analytically integrate the number of biomarkers and complex interrelationships among them is recognized in the literature (2).

Recent advances in biodemographic models of aging resulted in development of mathematical models of aging that are based on biological theory, incorporate several essential mechanisms of aging-related changes in organisms, and work with relevant socio-demographic and other information collected in longitudinal data on aging. These models, known as the stochastic process models (SPM) or the quadratic hazard models (4, 8–10), allow us to evaluate “hidden components” of aging-related changes including adaptive capacity, resistance to stresses, physiological norms, and effects of allostatic adaptation – all variables that play important roles in the processes of aging (11–15); their inclusion in the model is therefore important to understand regulatory mechanisms driving observed aging-related changes in physiological variables and their influence on risks of death or developing a chronic disease, as well as for evaluating a genetic component in such processes. The advantage of the SPM-based approaches is that “hidden components of aging” can be estimated from the data although corresponding variables are not directly observed in the study.

Recently, Cohen et al. (5) presented a novel approach for measuring physiological dysregulation via the joint distribution of multiple biomarkers based on calculation of a multivariate distance called the Mahalanobis distance (which we denote *D*_M_) (16, 17). Such a measure represents the deviation of a current physiological state of an organism from the “normal” physiological state. In contrast to previous approaches, such as those used to calculate allostatic load, *D*_M_ does not depend on a choice of biomarkers known to be associated with poor health, and thus avoids a potential for circularity (18). The interpretation of *D*_M_ as physiological dysregulation has been validated by showing that it (a) increases with age within individuals, (b) predicts mortality controlling for age, (c) gains predictive power as more variables are included, and (d) is not particularly sensitive to which markers are included, nor to their individual correlations with age (5). It has even been shown to function in a wild bird species (19). While individual trajectories of *D*_M_ with age have been examined in relation to health outcomes (20), the modeling approach used lacked the advantages of SPMs listed above, notably the ability to quantify adaptive capacity and optimized versus realized trajectories. Differences between men and women have also not been characterized.

The primary goal of this paper is to understand individual trajectories of dysregulation during aging in terms of adaptive capacity, mortality risk, and male–female differences. To do this, we will implement the measure of multivariate distance (*D*_M_) in the SPM framework. Joining the two approaches (SPM, *D*_M_) in the same model can further increase our capacity to uncover important aging-related processes. First, *D*_M_ can be used as a quantitative measurement of how an individual departs from the physiological norm and is thus directly informative about parameters to be estimated by SPM. Second, although successfully applied to different data in different settings in one- or two-dimensional cases (3, 4, 21–27), the SPM may face computational difficulties in applications to multidimensional data because of a large number of parameters to be estimated and a computationally intensive likelihood maximization procedure. *D*_M_ is useful in application of the SPM because it allows us to work with multiple physiological variables in a one-dimensional model while still allowing us to estimate and interpret all components of this one-dimensional SPM in the same way as in the original model. The SPM originates from the Woodbury–Manton model (28) and complexities associated with multidimensional versions of such models are recognized, see, e.g., Ref. (29), so the current approach is an important step forward in this research area. This paper is the first one addressing the combined effect of multiple physiological variables on mortality in the SPM (8) framework. We carry this out by applying the model to the Framingham Original Cohort (FHS) data. We estimate the model separately for female and male FHS participants to investigate sex differences in the dynamic behavior of the measure of physiological dysregulation and its possible relationships to the observed sex-specific differences in mortality risks.

## Data and Methods

### Measure of Statistical Distance and Physiological Dysregulation

For a given set of physiological variables (biomarkers) represented by a column vector *X* measured in an individual at age *t*, *X*(*t*), *D*_M_ is defined as (5):

(1) $$D_{M}(X(t))=\sqrt{{\left(X(t)-\bar{X}\right)}^{T}S^{-1}(X(t)-\bar{X})}$$

where $\bar{X}$ is a vector of means, *S* is the variance–covariance matrix for respective variables calculated from some population defining the “normal” state (which can be the same population or some other “reference” population), and *T* denotes transposition.

### Mathematical Model of Age Dynamics of Physiological Variables, Aging, and Mortality

Recent advances in mathematical modeling of aging allow us to link the age dynamics of physiological variables and mortality risks and evaluate “hidden components” of aging-related changes incorporating several major concepts of aging in the structure of the model. These models, originating from the Woodbury–Manton’s random walk model (28), became known as the SPM (8), see Section “Introduction.” Theoretical background of the models with survival functions induced by stochastic covariates is presented in Ref. (28, 30, 31). These results are directly applicable to the SPM version by Yashin et al. (8). The random evolution of covariates with age or time (which is a natural assumption, for example, for the evolution of biomarkers affecting survival) is not explicitly modeled in standard approaches, such as logistic or Cox models, and it is captured in such stochastic models. This is a fundamental feature of such models, which is an appealing property for practical applications, but the presence of stochastic covariates requires computations of marginal hazards and survival functions from the conditional hazards. When the conditional hazard is a quadratic function of such random covariates [as in Ref. (8)], such expressions can be computed as shown in Ref. (32, 33). Below, we briefly describe the SPM (8) in which we implement the measure of statistical distance (Eq. 1).

Briefly, the SPM consists of two equations representing the dynamics of chosen variables as a function of age *t* and conditional mortality rate at age *t* given the vector of the variables measured at respective ages. Let *Y*(*t*) be a *K*-dimensional stochastic process representing a (*K* × 1) vector of variables at age *t*. The conditional hazard of death is specified as:

(2) $$\mu \;(t,\;Y(t))=\mu_{0}(t)+{\left(Y(t)-f_{0}(t)\right)}^{T}Q(t)\;(Y(t)-f_{0}(t))$$

Here, μ_0_(*t*) is the “residual” or “baseline” hazard that represents the mortality risk, which would remain if the variables in *Y*(*t*) followed the “optimal trajectory” represented by a (*K* × 1) vector function *f*_0_(*t*). This baseline hazard μ_0_(*t*) models the effect of other risk factors not included in *Y*(*t*) that impact the risk of death. The age trajectory of variables *f*_0_(*t*) that minimizes the risk of death is referred to as the “physiological norm.” The matrix *Q*(*t*) is a (*K* × *K*) positive-definite symmetric matrix.

The dynamics of the stochastic process *Y*(*t*) is given by the following equation:

(3) $$dY(t)=a\;(t)\;(Y(t)-f_{1}(t))\;dt+b(t)\;dW(t)$$

with initial condition *Y*(*t*_0_). Here, *W*(*t*) is a vector Wiener process with independent components [*W*(*t*) is assumed to be independent of the initial vector *Y*(*t*_0_)], which defines random paths of the variables in *Y*(*t*). A matrix of diffusion coefficients *b*(*t*) regulates how this “randomness” propagates to variability of trajectories of *Y*(*t*).

The vector function *f*_1_(*t*) (referred to as the “mean allostatic state”) describes the effect of allostatic adaptation (13). This state characterizes the allostatically modified set point for homeostatic regulation, that is, the physiological state that organisms are forced to maintain by the process of homeostatic adaptation, which represents averaged effects of complex interplay among factors controlled by the ontogenetic program, senescence, and long-term stresses exceeding the limits of the organism’s homeostatic regulation. This dynamic behavior [i.e., that *Y*(*t*) adapts to changes in the function *f*_1_(*t*)] in the model is possible due to the presence of the negative feedback mechanism in Eq. 3 with coefficients of homeostatic regulation given by a matrix *a*(*t*). Age-related changes in these coefficients characterize changes in adaptive capacity with age. Specifically, the elements of this matrix characterize the rate of the adaptive response for any deviation of the variables from the state *f*_1_(*t*). This allows us to test hypotheses on decline in adaptive capacity with age [see also more discussion and examples in Ref. (8, 10)]. Note that the general model with age-dependent components in Eqs 2 and 3 can be simplified to the version with age-independent components. This may be helpful in some applications where testing the hypotheses on age-dependence of respective characteristics is not important but in our applications age-dependence of all components [except *f*_0_(*t*) and *b*(*t*)] is essential as it allows for addressing the hypotheses formulated below in Section “Application to Framingham Heart Study Data.”

The SPM described above can be applied to any set of variables for which longitudinal measurements are available in the data. The measure of physiological dysregulation (*D*_M_) represents an example of such a variable: it can be calculated for each individual at each observation point (exam) so that each individual has a trajectory of measurements of *D*_M_ at different ages.

### Mathematical Model of Age Trajectories of Physiological Dysregulation, Aging, and Mortality

Let *Y*(*t*) = *D*_M_[*X*(*t*)] as given by Eq. 1. This is now a one-dimensional process represented by Eq. 3 where respective components [*W*(*t*), *f*_1_(*t*), *a*(*t*), *b*(*t*)] are scalar counterparts of corresponding vectors/matrices. The one-dimensional version of Eq. 2 is:

(4) $$\mu \;(t,\;Y(t))=\mu_{0}(t)+Q(t)\;{\left(Y(t)-f_{0}(t)\right)}^{2}$$

where *f*_0_(*t*) and *Q*(*t*) are also scalars. As *D*_M_ measures physiological dysregulation in an organism represented as deviations from the “normal state” (as specified in its calculation), it is natural to assume that *f*_0_(*t*) = 0 in Eq. 4. That is, in this implementation we assume that the “normal” values of physiological variables from the definition of *D*_M_ (Eq. 1) minimize the risk of death at all ages [see also Section “Results and Discussion” regarding the specification of the “normal state” in *D*_M_ calculations and alternative implementations of the “norm” *f*_0_(*t*)]. Note that the definition of *D*_M_ involves calculations of a vector of means and the variance–covariance matrix for respective variables from some “reference” population defining the “normal” state. The question of which “reference” population should be used for these purposes can be dictated by data availability and research paradigms in specific studies, but the results are generally not overly sensitive to specifics of the choice (34). Reference populations that differ markedly from study populations across many demographic aspects (e.g., age, race, and sex) perform poorly, but differences in any single aspects have mostly minor effects. In our application, we used the population of individuals from the original Framingham cohort aged 40 years and younger at the baseline exam, as described below in Section “Application to Framingham Heart Study Data.”

### Application to Framingham Heart Study Data

The Framingham Original Cohort was launched in 1948 and has continued with biennial examinations to the present. The FHS Original Cohort included 5,209 respondents (nearly all subjects were Caucasians) aged 28–62 years at baseline residing in Framingham, MA, USA, between 1948 and 1951. Data on 5,079 individuals (2,785 females, 2,294 males) from the Original Cohort were available for this study. Data relevant to our analyses include ages at exams, sex, ages at death/censoring (available data contain information on the number of days since the first exam until the event/censoring from which we calculated respective ages dividing the number of days by 365.25), and various physiological variables measured at exams. For our analyses, we selected physiological variables measured at a sufficient number of exams and whose dynamic characteristics have been shown to be related to the risk of death or onset of aging-related diseases in participants of the FHS Original Cohort in our earlier studies including those which used the original SPM approach [see, e.g., Ref. (3, 4, 24, 26, 27, 35)]. The list includes blood glucose (BG), body mass index (BMI), total cholesterol (CH), diastolic blood pressure (DBP), hematocrit (HC), systolic blood pressure (SBP), pulse pressure (PP), and ventricular rate (VR). Their relevance to research on aging has been discussed in the literature, see, e.g., an earlier publication by Manton et al. (36).

The original data on physiological measurements contain missing values (either by design when at some exam the respective variable was not measured or intermittent missing values when some individuals missed an exam). Following Engels and Diehr (37), we imputed intermittent missing longitudinal values for an individual using available data for that individual. Intermittent missing values were imputed using a linear approximation of available observations in the adjacent exams. For missing data at initial exams, two methods were used: the “next observation carried backward” method and the average of measurements at the next two exams. We repeated all computations using the data generated by these two methods and the results were not sensitive to the imputation method so only the results that used the latter method are reported here.

The data on original variables (BG, BMI, CH, DBP, HC, PP, SBP, and VR) were each transformed using the Box–Cox transformation and then standardized to a zero mean and a unit variance so that all transformed variables are on the same scale in the analyses. We used these standardized transformed variables in calculations of *D*_M_, as defined in Eq. 1. We calculated the means and the variance–covariance matrix in Eq. 1 using baseline measurements for individuals aged 40 years and younger (altogether, there were 2,012 such individuals in the Original Cohort, 1,104 females and 908 males). All analyses were performed separately for females and males (to avoid any assumptions on relationships between parameters of the model in two sexes) using the sets of physiological variables included in calculations of *D*_M_ variants, as described below.

The values of *D*_M_ depend on the specific set of variables included in its definition. Therefore, to test if the results are sensitive to the choice of variables in *D*_M_, it is necessary to perform analyses with different variants of *D*_M_ based on different subsets of variables. The most comprehensive approach would be to use all possible combinations of variables as in Cohen et al. (5). However, as the present study applies the SPM that involves intensive computations, the analyses of all such variants are not feasible. Therefore, we selected a “basic” variant of *D*_M_ and performed sensitivity analyses for an additional (limited) set of variants to test whether the results are sensitive to the choice of the variables in the construction of *D*_M_. Our “basic” variant is “the most complete” *D*_M_ when all variables are included in its construction. Note that, as PP = SBP − DBP, we cannot use these three variables simultaneously in analyses. Therefore, we considered three “basic” variants each including seven variables: BG, BMI, CH, HC, VR, and two variables out of DBP, PP, and SBP. We also computed several additional variants of *D*_M_ to perform sensitivity analyses to test: (a) whether the removal of one variable from such “basic” variants substantially changes the results; and (b) whether the removal of HC and VR (which, unlike the other variables, were not measured until exam 4) from such “basic” variants substantially changes the results. Altogether, we analyzed 24 different *D*_M_ variants.

In our applications of SPM (Eqs 3 and 4) to these *D*_M_ variants we used the following functional forms of the model’s components: (1) a linear function of age for the feedback coefficient: *a*(*t*) = *a_*Y*_* + *b_*Y*_*(*t* − *t*_min_), where *a_*Y*_* < 0, *b_*Y*_* ≥ 0, and *t*_min_ = 28 (which is the minimal age at the first exam in the FHS Original Cohort); (2) a linear function of age for the mean allostatic trajectory: $f_{1}(t)=a_{f_{1}}+b_{f_{1}}(t-t_{\min})$; (3) a linear function of age for the multiplier in the quadratic hazard term [named “vulnerability index” in Arbeev et al. (4) as it characterizes the “robustness,” or “vulnerability,” a component of stress resistance]: *Q*(*t*) = *a_*Q*_* + *b_*Q*_*t**; (4) the Gompertz baseline hazard: $\ln\mu_{0}(t)=\ln a_{\mu_{0}}+b_{\mu_{0}}(t-t_{\min})$; (5) a constant diffusion coefficient: *b*(*t*) = σ_1_; and (6) a normally distributed initial value $Y(t_{0})\sim N(f_{1}(t_{0}),\sigma_{0}^{2})$.

This is a parsimonious specification of the model that still allows us to test several hypotheses on the impact of systemic dysregulation on mortality risk and its relation to different aging-related processes represented by components of the model:

(1) H0: *Q*(*t*) = 0 [i.e., *a_*Q*_* = 0 and *b_*Q*_* = 0, so that there is no quadratic term in the hazard rate (Eq. 4) and mortality is described by the baseline Gompertz rate μ_0_(*t*)]. This is the most important hypothesis because the failure to reject this hypothesis would not allow us to claim that *D*_M_ is a legitimate characteristic for capturing physiological dysregulation affecting mortality risk in this sample.
(2) H0: *Q*(*t*) = *a_*Q*_* (i.e., *b_*Q*_* = 0). Rejection of this hypothesis would mean that the effect of physiological dysregulation on mortality risk is age dependent. This hypothesis allows us to make inferences on aging-related change in stress resistance associated with the variables used in *D*_M_. For example, if *Q*(*t*) increases with age then the J-shape of the hazard rate (as a function of *D*_M_ at a fixed age *t*) narrows with age indicating that the same value of *D*_M_ induces a larger increase in mortality risk at old ages compared to younger ages, which can be interpreted as the aging-related decline in resistance to stresses associated with the dynamics of the respective variables, see Yashin et al. (8) and Arbeev et al. (4).
(3) H0: *f*_1_(*t*) = 0 (i.e., $a_{f_{1}}=0$ and $b_{f_{1}}=0$). This hypothesis allows us to make inferences on the presence of a systemic dysregulation for the specific set of variables used in *D*_M_. Rejection of this hypothesis would mean that there is a systemic dysregulation in an organism that forces the trajectories of physiological variables to deviate from their “normal values” specified in calculation of *D*_M_ [or, equivalently, forces *Y*(*t*) to deviate from *f*_0_(*t*)].
(4) H0: $f_{1}(t)=a_{f_{1}}$ (i.e., $b_{f_{1}}=0$). This hypothesis allows us to make inferences on the changes in the level of systemic dysregulation with age for the specific set of variables used in *D*_M_. Rejection of this hypothesis would mean that the level which the trajectory of *Y*(*t*) is forced to follow changes with age. For example, increasing *f*_1_(*t*) with age would mean an increasing level of systemic physiological dysregulation with age (so that the trajectories of physiological variables deviate further with age from their “norms” defined at younger ages), which can be associated with the manifestation of the aging process.
(5) H0: *a*(*t*) = *a_*Y*_* (i.e., *b_*Y*_* = 0). This hypothesis allows us to make inferences on the decline in adaptive capacity with age for the specific set of variables used in *D*_M_. Rejection of this hypothesis would mean that the value of the feedback coefficient in Eq. 3 changes with age. As noted, this feedback coefficient in the model is associated with the adaptive capacity of an organism [i.e., the rate of the adaptive response for any deviation of *Y*(*t*) from *f*_1_(*t*)]. In general, the larger the absolute value of *a*(*t*), the faster *Y*(*t*) tends to *f*_1_(*t*). This means that if the absolute value of *a*(*t*) declines with age then more time is needed for the trajectory of *Y*(*t*) to go back to *f*_1_(*t*) at old ages compared to younger ages, which is the manifestation of the decline in adaptive capacity with age. Note also that *Y*(*t*) can deviate from *f*_1_(*t*) in both directions, i.e., closer to zero as well as toward larger values, and if the absolute value of *a*(*t*) declines with age then such periods when the trajectory of *D*_M_ departs farther from *f*_1_(*t*) tend to be longer at old ages. This may mean that aging-related changes observed in the aging human body may also manifest effects of compensatory adaptation which tend to reduce dysregulation effects when *f*_1_(*t*) becomes large enough. These considerations stimulate further development of methods of dynamic modeling of aging-related changes and their connections with health and survival outcomes. See also further discussion on relationships between adaptive capacity, dysregulation and different components of aging-related changes in Section “Results and Discussion.”

Testing the above hypotheses involves fitting the restricted models (with restrictions on the parameters specified as in the parentheses above) along with the original (unrestricted) model using the same likelihood estimation procedure (8). As we deal with nested models in all cases, the likelihood ratio test can be used to make statistical inference (see Table S1 in Supplementary Material). The likelihood maximization in the SPM is performed using the constrained optimization procedure (implemented, e.g., in the MATLAB’s Optimization toolbox). Constrained optimization is needed because constraints on parameter values are necessary in respective components of the model both for the mortality risk as well as for the risk factor *Y*(*t*). The details can be found elsewhere [e.g., Ref. (8, 9)]. Also note that *Y*(*t*) can always be transformed (e.g., using the logarithm or the Box–Cox transformation) similar to the original variables *X*(*t*), if necessary. The original scale was used in the paper for the sake of interpretability of the resulting trajectories.

## Results and Discussion

### Estimates of Parameters and Results of Testing Hypotheses

Estimates of parameters of the SPM applied to different variants of *D*_M_ calculated for individuals from the Framingham Original Cohort as described in Section “Application to Framingham Heart Study Data” are given in Table 1 (for females) and Table 2 (for males). The tables show that, although the parameter estimates differ for different variants of *D*_M_, they still follow a common pattern so that generally the components of the model look similar in applications to considered *D*_M_ variants (a few exceptions are described below). The tables also reveal systematic differences between estimates of parameters in females and males. This is addressed in more detail in Section “Sex-Specific Differences in Estimates in the Model.”

**Table 1:** **Estimates of parameters of the stochastic process model applied to different variants of *D*_M_ calculated for females from the Framingham Original Cohort**.

*D*_M_ variables	Parameters^a,b^										ln L^c^
	$lna_{{µ}_{0}}$	$b_{{µ}_{0}}$	*a_Q_*· 10^4^	*b_Q_*· 10^5^	*a_Y_*	*b_Y_*· 10^3^	σ_0_	σ_1_	$a_{f_{1}}$	$b_{f_{1}}$	
BG, BMI, CH, DBP, HC, SBP, VR	−13.86	0.172	−0.520^§^	0.186^#^	−0.178	0.832^†^	0.97	0.58	2.06^†^	0.055^†^	−51646.89
BG, BMI, CH, DBP, HC, PP, VR	−13.73	0.170	−0.522^§^	0.186^#^	−0.179	0.765^†^	1.01	0.55	2.20^†^	0.046^†^	−49857.15
BG, BMI, CH, HC, PP, SBP, VR	−13.72	0.170	−0.535^§^	0.191^#^	−0.191	1.027^†^	0.95	0.53	2.24^†^	0.043^†^	−48934.60
BG, BMI, CH, DBP, SBP	−12.87	0.155	−0.490^†^	0.260*	−0.177	0.981^†^	0.94	0.60	1.53^†^	0.060^†^	−61306.99
BG, BMI, CH, DBP, PP	−13.27	0.163	−0.853^†^	0.350^#^	−0.185	1.154^†^	0.98	0.54	1.70^†^	0.049^†^	−57547.70
BG, BMI, CH, PP, SBP	−13.44	0.166	−1.003^†^	0.392^#^	−0.198	1.369^†^	0.92	0.51	1.79^†^	0.042^†^	−55461.22
BMI, CH, DBP, HC, SBP, VR	−12.24	0.137	−1.054^†^	0.376^#^	−0.161	0.328	0.95	0.57	1.88^†^	0.054^†^	−53324.65
BMI, CH, DBP, HC, PP, VR	−12.57	0.145	−1.126^†^	0.402^#^	−0.160	0.206	0.99	0.53	2.03^†^	0.045^†^	−51264.65
BMI, CH, HC, PP, SBP, VR	−12.59	0.146	−1.158^†^	0.414^#^	−0.173	0.480*	0.94	0.52	2.08^†^	0.041^†^	−50236.56
BG, CH, DBP, HC, SBP, VR	−11.79	0.137	−1.250^†^	0.447^#^	−0.197	1.227^†^	0.96	0.60	1.88^†^	0.055^†^	−58255.78
BG, CH, DBP, HC, PP, VR	−12.02	0.142	−1.345^†^	0.480^§^	−0.197	1.135^†^	1.00	0.56	2.02^†^	0.046^†^	−56406.84
BG, CH, HC, PP, SBP, VR	−12.14	0.144	−1.403^†^	0.501^§^	−0.210	1.388^†^	0.95	0.55	2.05^†^	0.043^†^	−55579.67
BG, BMI, DBP, HC, SBP, VR	−12.56	0.162	−0.676^†^	0.242^§^	−0.170	0.525^#^	1.00	0.61	1.74^†^	0.058^†^	−61075.97
BG, BMI, DBP, HC, PP, VR	−12.44	0.161	−0.655^§^	0.234^#^	−0.171	0.426*	1.03	0.56	1.89^†^	0.048^†^	−58788.66
BG, BMI, HC, PP, SBP, VR	−12.40	0.160	−0.657^§^	0.235^#^	−0.185	0.697^§^	0.97	0.55	1.93^†^	0.044^†^	−57709.21
BG, BMI, CH, HC, SBP, VR	−13.33	0.164	−0.556^§^	0.199*	−0.156	0.204	0.95	0.51	2.19^†^	0.034^†^	−47813.00
BG, BMI, CH, HC, PP, VR	−13.79	0.172	−0.598^§^	0.213^#^	−0.181	0.899^†^	0.92	0.53	1.98^†^	0.046^†^	−48462.89
BG, BMI, CH, DBP, SBP, VR	−13.13	0.160	−0.750^†^	0.268^#^	−0.176	0.908^†^	0.96	0.61	1.74^†^	0.062^†^	−61663.48
BG, BMI, CH, DBP, PP, VR	−13.37	0.165	−0.842^†^	0.301^§^	−0.181	0.907^†^	0.99	0.55	1.93^†^	0.050^†^	−58231.94
BG, BMI, CH, PP, SBP, VR	−13.36	0.165	−0.868^†^	0.310^§^	−0.190	0.979^†^	0.93	0.53	2.00^†^	0.044^†^	−56720.57
BG, BMI, CH, DBP, HC, VR	−13.50	0.167	−0.657^§^	0.235^#^	−0.162	0.000	0.92	0.50	2.27^†^	0.023^†^	−46868.24
BG, BMI, CH, DBP, HC, SBP	−13.40	0.163	−0.560^§^	0.200^#^	−0.180	0.925^†^	0.96	0.57	1.86^†^	0.053^†^	−51207.11
BG, BMI, CH, DBP, HC, PP	−13.32	0.163	−0.563^§^	0.201^#^	−0.182	0.963^†^	0.99	0.54	1.99^†^	0.045^†^	−49271.98
BG, BMI, CH, HC, PP, SBP	−13.34	0.163	−0.585^§^	0.209^#^	−0.196	1.293^†^	0.94	0.52	2.04^†^	0.040^†^	−48112.82

*^a^The estimates of some parameters are rescaled for better visibility in the table: *a_Q_* is multiplied by 10^4^; *b_Q_* is multiplied by 10^5^; and *b_Y_* is multiplied by 10^3^*.

*^b^The symbols after the numbers in the following columns of table denote *p*-values (evaluated by the likelihood ratio test) for different null hypotheses: column “*a_Q_*⋅10^4^”: null hypothesis – zero quadratic part of the hazard, i.e., *Q*(*t*) = 0 (*a_Q_* = 0 and *b_Q_* = 0); column “*b_Q_*⋅10^5^”: null hypothesis – age-independent J-shape of the hazard, i.e., *Q*(*t*) = *a_Q_* (*b_Q_* = 0); column “*b_Y_*⋅10^3^”: null hypothesis – no aging-related decline in the adaptive capacity, i.e., *a*(*t*) = *a_Y_* (*b_Y_* = 0); column “$a_{f_{1}}$”: null hypothesis – no systemic dysregulation in an organism, i.e., *f*_1_(*t*) = 0 ($a_{f_{1}}=0$ and $b_{f_{1}}=0$); and column “$b_{f_{1}}$”: null hypothesis – age-independent level of systemic physiological dysregulation, i.e., $f_{1}(t)=a_{f_{1}}$ ($b_{f_{1}}=0$)*.

The symbols in these columns denote: ^†^*p* < 0.0001; ^§^0.0001 ≤ *p* < 0.001; ^#^0.001 ≤ *p* < 0.01; and *0.01 ≤ *p* < 0.05, for respective null hypotheses. The absence of symbols after the numbers in these columns means that respective *p*-values exceed 0.05. Note that all other columns in the table, except the columns mentioned above, are not used to represent information on testing any null hypotheses and therefore they do not contain any symbols.

*^c^ln L, logarithm of the likelihood function*.

**Table 2:** **Estimates of parameters of the stochastic process model applied to different variants of *D*_M_ calculated for males from the Framingham Original Cohort**.

*D*_M_ variables	Parameters^a,b^										ln L^c^
	$\ln\;a_{{µ}_{0}}$	$b_{{µ}_{0}}$	*a_Q_* · 10^4^	*b_Q_* · 10^5^	*a_Y_*	*b_Y_* · 10^3^	σ_0_	σ_1_	$a_{f_{1}}$	$b_{f_{1}}$	
BG, BMI, CH, DBP, HC, SBP, VR	−12.00	0.157	−1.901^†^	0.679^†^	−0.176	0.072	0.87	0.54	2.23^†^	0.031^†^	−39006.26
BG, BMI, CH, DBP, HC, PP, VR	−11.98	0.157	−1.890^†^	0.675^†^	−0.192	0.284	0.88	0.53	2.32^†^	0.027^†^	−38742.16
BG, BMI, CH, HC, PP, SBP, VR	−11.87	0.155	−1.862^†^	0.665^†^	−0.208	0.578*	0.84	0.53	2.35^†^	0.024^†^	−38455.61
BG, BMI, CH, DBP, SBP	−10.65	0.128	−3.665^†^	1.309^†^	−0.211	1.747^†^	0.86	0.52	1.70^†^	0.034^†^	−47230.85
BG, BMI, CH, DBP, PP	−10.76	0.130	−3.871^†^	1.382^†^	−0.233	2.064^†^	0.86	0.50	1.80^†^	0.028^†^	−46222.86
BG, BMI, CH, PP, SBP	−10.72	0.130	−3.934^†^	1.405^†^	−0.249	2.221^†^	0.82	0.49	1.87^†^	0.023^†^	−45581.04
BMI, CH, DBP, HC, SBP, VR	−11.55	0.146	−3.214^†^	1.148^†^	−0.171	0.000	0.85	0.53	2.05^†^	0.030^†^	−42764.52
BMI, CH, DBP, HC, PP, VR	−11.54	0.147	−3.209^†^	1.146^†^	−0.181	0.000	0.86	0.52	2.14^†^	0.025^†^	−42432.30
BMI, CH, HC, PP, SBP, VR	−11.46	0.146	−3.202^†^	1.144^†^	−0.190	0.000	0.82	0.52	2.18^†^	0.022^†^	−42085.78
BG, CH, DBP, HC, SBP, VR	−10.32	0.125	−3.839^†^	1.371^†^	−0.190	0.209	0.87	0.55	2.03^†^	0.031^†^	−44353.45
BG, CH, DBP, HC, PP, VR	−10.47	0.129	−3.946^†^	1.409^†^	−0.205	0.359	0.88	0.54	2.12^†^	0.027^†^	−44027.24
BG, CH, HC, PP, SBP, VR	−10.38	0.127	−3.906^†^	1.395^†^	−0.220	0.564*	0.85	0.54	2.15^†^	0.024^†^	−43794.58
BG, BMI, DBP, HC, SBP, VR	−11.39	0.157	−2.370^†^	0.846^†^	−0.183	0.219	0.88	0.55	1.96^†^	0.035^†^	−46361.15
BG, BMI, DBP, HC, PP, VR	−11.34	0.157	−2.325^†^	0.830^†^	−0.195	0.231	0.88	0.54	2.05^†^	0.030^†^	−45917.42
BG, BMI, HC, PP, SBP, VR	−11.23	0.155	−2.238^†^	0.799^†^	−0.209	0.409	0.84	0.54	2.09^†^	0.027^†^	−45565.79
BG, BMI, CH, HC, SBP, VR	−11.74	0.154	−2.053^†^	0.733^†^	−0.177	0.000	0.84	0.51	2.23^†^	0.019^†^	−37438.01
BG, BMI, CH, HC, PP, VR	−11.59	0.151	−1.970^†^	0.703^§^	−0.197	0.538*	0.83	0.52	2.13^†^	0.024^†^	−38109.39
BG, BMI, CH, DBP, SBP, VR	−10.86	0.133	−2.640^†^	0.943^†^	−0.200	1.157^†^	0.87	0.54	1.92^†^	0.036^†^	−47889.24
BG, BMI, CH, DBP, PP, VR	−10.92	0.135	−2.714^†^	0.969^†^	−0.214	1.224^†^	0.87	0.53	2.03^†^	0.030^†^	−47136.73
BG, BMI, CH, PP, SBP, VR	−10.83	0.134	−2.686^†^	0.959^†^	−0.229	1.354^†^	0.83	0.52	2.09^†^	0.027^†^	−46628.07
BG, BMI, CH, DBP, HC, VR	−11.85	0.155	−2.283^†^	0.815^†^	−0.177	0.000	0.82	0.50	2.18^†^	0.019^†^	−37076.87
BG, BMI, CH, DBP, HC, SBP	−11.79	0.153	−2.167^†^	0.774^†^	−0.180	0.355	0.87	0.53	2.03^†^	0.029^†^	−38428.67
BG, BMI, CH, DBP, HC, PP	−11.77	0.153	−2.148^†^	0.767^†^	−0.198	0.653^#^	0.87	0.52	2.12^†^	0.025^†^	−38128.94
BG, BMI, CH, HC, PP, SBP	−11.68	0.152	−2.128^†^	0.760^†^	−0.215	0.926^§^	0.84	0.51	2.16^†^	0.021^†^	−37779.68

*^a^The estimates of some parameters are rescaled for better visibility in the table: *a_Q_* is multiplied by 10^4^; *b_Q_* is multiplied by 10^5^; *b_Y_* is multiplied by 10^3^*.

*^b^The symbols after the numbers in the following columns of Table 1 denote *p*-values (evaluated by the likelihood ratio test) for different null hypotheses: column “*a_Q_*⋅10^4^”: null hypothesis – zero quadratic part of the hazard, i.e., *Q*(*t*) = 0 (*a_Q_* = 0 and *b_Q_* = 0); column “*b_Q_*⋅10^5^”: null hypothesis – age-independent J-shape of the hazard, i.e., *Q*(*t*) = *a_Q_* (*b_Q_* = 0); column “*b_Y_*⋅10^3^”: null hypothesis – no aging-related decline in the adaptive capacity, i.e., *a*(*t*) = *a_Y_* (*b_Y_* = 0); column “$a_{f_{1}}$”: null hypothesis – no systemic dysregulation in an organism, i.e., *f*_1_(*t*) = 0 ($a_{f_{1}}=0$ and $b_{f_{1}}=0$); and column “$b_{f_{1}}$”: null hypothesis – age-independent level of systemic physiological dysregulation, i.e., $f_{1}(t)=a_{f_{1}}$ ($b_{f_{1}}=0$)*.

The symbols in these columns denote: ^†^*p* < 0.0001; ^§^0.0001 ≤ *p* < 0.001; ^#^0.001 ≤ *p* < 0.01; and *0.01 ≤ *p* < 0.05, for respective null hypotheses. The absence of symbols after the numbers in these columns means that respective *p*-values exceed 0.05. Note that all other columns in the table, except the columns mentioned above, are not used to represent information on testing any null hypotheses and therefore they do not contain any symbols.

*^c^ln L, logarithm of the likelihood function*.

Tables 1 and 2 also present the results of the hypothesis tests specified in Section “Application to Framingham Heart Study Data” (see notes in the tables for definitions of symbols or the absence of those used in different columns of the tables). Most importantly, the tables show that the null hypotheses H0: *Q*(*t*) = *0* is rejected for all variants of *D*_M_ (*p* < 0.0001 for all *D*_M_ in males and most *D*_M_ in females, with maximal *p* = 0.0007 for *D*_M_ with BG, BMI, CH, HC, SBP, and VR). This allows us to conclude that there is a quadratic term in the hazard rate (Eq. 4) so that mortality is not entirely captured by the baseline Gompertz rate μ_0_(*t*), regardless of the variant of *D*_M_. This means that *D*_M_ captures effects of deviations of physiological variables on mortality in this application. All considered *D*_M_ affect mortality risk in this sample so that non-zero values of *D*_M_ result in a higher mortality rate compared to the baseline mortality rates at respective ages. Note also that, although we have non-zero estimates of the multiplier *Q*(*t*) and the quadratic term is present in Eq. 4, we also have non-zero estimates of the baseline mortality rate μ_0_(*t*). There are many more risk factors that affect mortality risk and which are not included in definitions of *D*_M_ and, correspondingly, in the quadratic part of the hazard. This “residual” mortality rate μ_0_(*t*) summarizes the effects of such unspecified factors affecting mortality risk.

The null hypothesis H0: *Q*(*t*) = *a_*Q*_* is also rejected in all cases, with *p* ≤ 0.0001 for males and *p*-values ranging between 0.0005 and 0.01 for females (except *D*_M_ with BG, BMI, CH, DBP, and SBP for which *p* = 0.038). Tables 1 and 2 also show that in all cases the parameter *b_*Q*_* is positive. These observations indicate that the effect of physiological dysregulation (represented by *D*_M_) on mortality risk is age dependent and that the J-shape of the mortality rate (as a function of *D*_M_ at any fixed age) narrows with age. This narrowing pattern with age has the effect that the same level of physiological dysregulation (i.e., the same value of *D*_M_) induces a larger increase in mortality risk at old ages than it does at younger ages (compared to the baseline mortality rate for respective age). This can be interpreted as the aging-related decline in resistance to stresses associated with deviant dynamics of respective physiological variables [see also Yashin et al. (8) and Arbeev et al. (4)].

The null hypotheses H0: *f*_1_(*t*) = 0 and H0: $f_{1}(t)=a_{f_{1}}$ are rejected in all cases (all *p* < 0.0001). This result shows the presence of a systemic dysregulation in an organism that forces the trajectories of physiological variables to deviate from their “normal values” specified in calculation of *D*_M_ (equivalently, forces *D*_M_ to deviate from zero). The effect of this dysregulation is that, on average, an individual tends to have the values of physiological variables that deviate from the “normal” state (i.e., he/she has non-zero *D*_M_) so that the resulting mortality risk is higher than it could be if that person managed to keep his/her physiological variables equal to the “norm” (i.e., to have zero *D*_M_) in which case the mortality risk would be equal to the baseline mortality at the respective age. Note also that in applications to all variants of *D*_M_ parameter $b_{f_{1}}$ is positive. This indicates an increasing level of systemic physiological dysregulation with age that manifests itself in trajectories of physiological variables drifting further away from their “norms” as age progresses.

The results for the null hypothesis H0: *a*(*t*) = *a_*Y*_* are mixed. For many variants of *D*_M_ (16 for females and 6 for males), the decline in the absolute value of the feedback coefficient *a*(*t*) with age (associated with the decline in adaptive capacity of an organism) is significant with *p* < 0.0001, and for some other variants, the decline is not significant, with some estimates *b_*Y*_* = 0. This means that the conclusions about the decline in adaptive capacity depend on the variables used in calculations of *D*_M_ and that different physiological variables have different behaviors in terms of the change with age in the ability of an organism to push them back to the trajectories specified by the mean allostatic trajectories. For those variables that indicated the decline in the absolute value of the feedback coefficient *a*(*t*) with age, this ability worsens with age and more time is needed for the trajectories of physiological variables to go back to their mean allostatic trajectories at old ages compared to the time needed at younger ages. However, for some physiological variables, this ability of an organism seems not to be affected by age. These results confirm our earlier observations on mixed patterns of age dynamics of adaptive capacity in analyses of different physiological variables and different outcomes (4, 26). A strong negative correlation between intercept and slope for the feedback coefficient *a*(*t*) (−0.86 for males, −0.92 for females) indicates that for the *D*_M_ variants with worse initial adaptive capacity (i.e., smaller absolute values of *a_*Y*_*) the subsequent decline tends to be slower than that for the *D*_M_ variants with better initial adaptive capacity (i.e., larger absolute values of *a_*Y*_*).

### Sex-Specific Differences in Estimates in the Model

**Figure 1:**
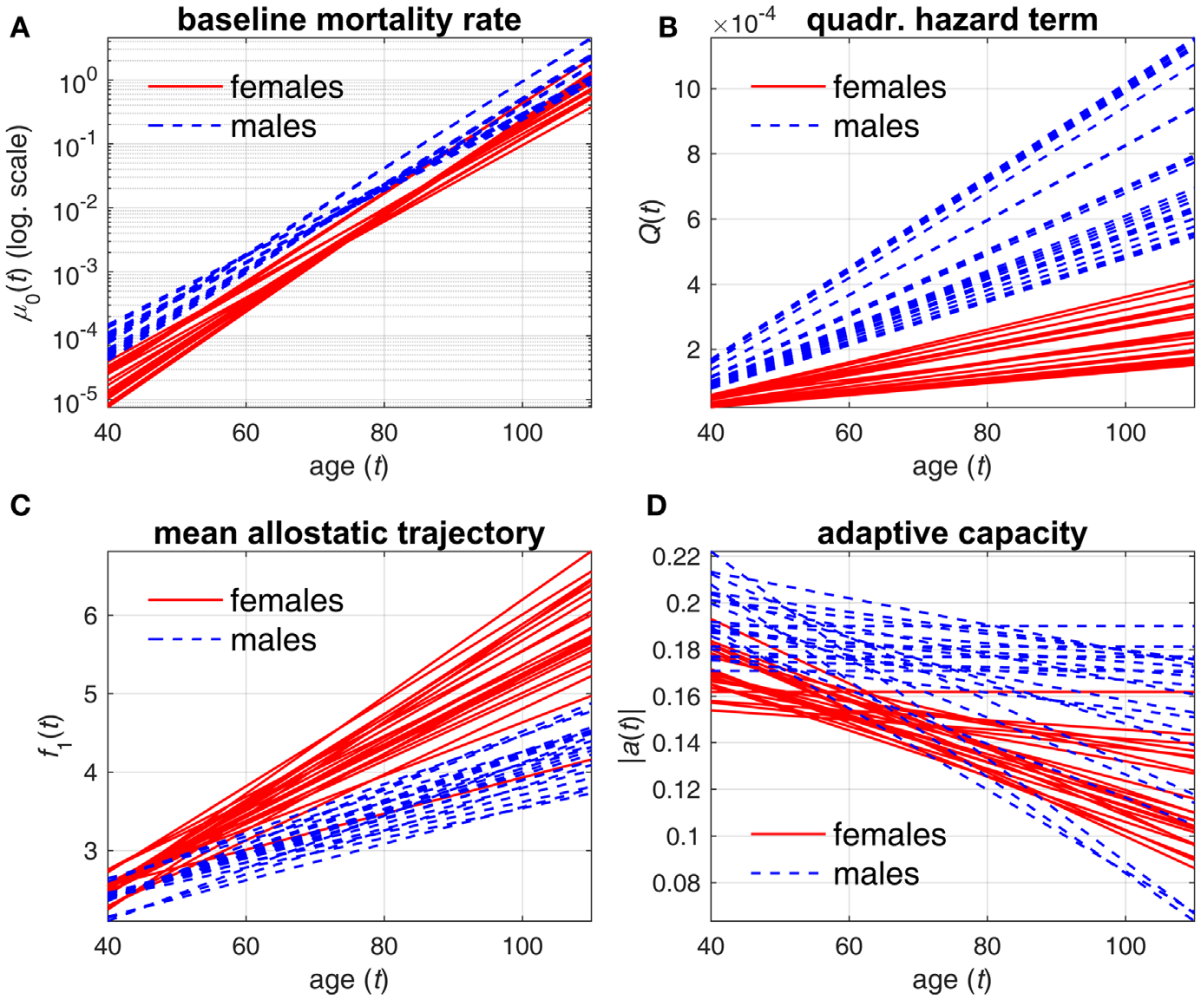
**Estimates of different components of stochastic process model applied to different variants of *D*_M_**. **(A)** baseline mortality rate μ_0_(*t*); **(B)** multiplier in quadratic hazard term *Q*(*t*); **(C)** mean allostatic trajectory *f*_1_(*t*); **(D)** absolute value of feedback coefficient *a*(*t*). Different lines correspond to estimates of respective components in specific *D*_M_ variants for females (solid lines) and males (dashed lines).

**Figure 2:**
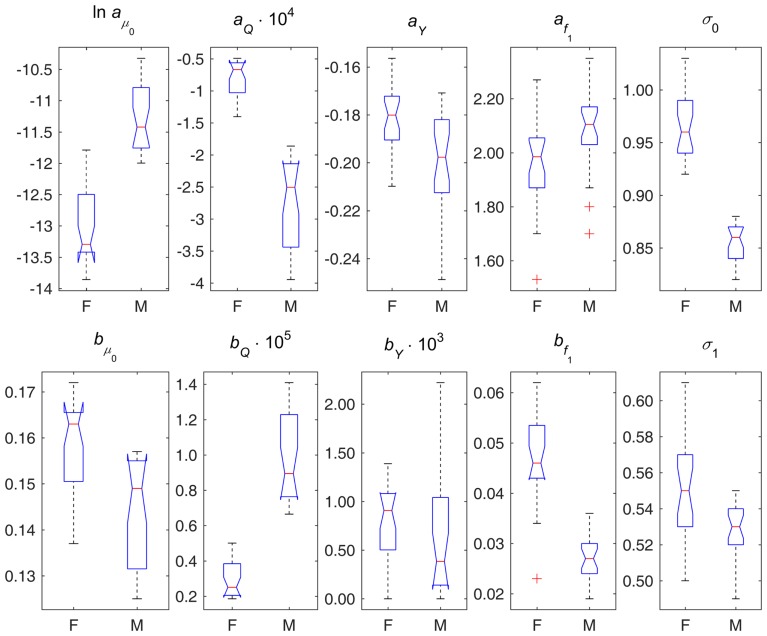
**Box plots of parameter estimates in different variants of *D*_M_ applied to data on females (F) and males (M)**. Note: points are shown as outliers (“+”) if they are larger than *q*_3_ + *w*(*q*_3_ − *q*_1_) or smaller than *q*_1_ − *w*(*q*_3_ − *q*_1_), where *q*_1_ and *q*_3_ are the 25th and 75th percentiles, respectively, and *w* = 1.5.

Tables 1 and 2 reveal systematic differences between estimates of parameters in females and males. These differences can be better understood from Figures 1 and 2. Figure 1 compares the estimates of female and male patterns of different components of the model applied to different variants of *D*_M_. It shows that the baseline mortality rate μ_0_(*t*) is higher in males. Males also have higher levels of the multiplier in the quadratic hazard term *Q*(*t*), which also increases faster with age than that in females. This means that males have a narrower J-shape of the mortality risk as a function of *D*_M_ at each age and, moreover, this J-shape narrows even further at a faster rate in males than the J-shape for females. This confirms our earlier observations (4) and suggests that males have generally lower resistance to stresses associated with deviant dynamics of the respective physiological variables than females: at each age males are more vulnerable to deviations from the “normal” state (i.e., the same value of *D*_M_ results in a larger increase in the mortality risk in males compared to females). Increasing patterns of *Q*(*t*) in both sexes show that the same value of deviations from the norm (i.e., the same value of *D*_M_) causes a larger increase in mortality rate (compared to the baseline rate at respective age) at old ages than it does at younger ages. This is true for both sexes but a faster increase of *Q*(*t*) with age in males implies that this “additional price” which an organism has to pay in terms of increasing mortality rate for deviations of physiological parameters from the “norm” increases faster with age in males than in females. This, along with a higher baseline mortality rate, results in the higher mortality rate for males that is observed in human populations.

Mean allostatic trajectories *f*_1_(*t*) show the opposite pattern – they are higher and increase faster in females. This indicates that the physiological variables summarized by the respective *D*_M_ tend to deviate farther from the “norm” in females and with age this gap widens faster in females. As such deviations generally correspond to abnormal values of physiological variables that are indicators of diseases/conditions (e.g., diabetes or hypertension), this can contribute to the observed higher prevalence of aging-related diseases and conditions in females. These results on sex differences agree with previous studies showing higher mortality in males but greater propensity for clinical frailty in females (38–40). However, the effect of this on mortality is attenuated by the observed lower values of the multiplier in the quadratic hazard term *Q*(*t*) in females compared to males. Nevertheless, for some compositions of *D*_M_, we can see no major differences in mean allostatic trajectories for females and males. This observation implies that for some specific indicators of health the male–female mortality–morbidity paradox (41, 42) may not hold and may even be reversed (43).

The results on the age dynamics of the feedback coefficient *a*(*t*) are mixed, although females have a tendency to have worse adaptive capacity (both initial adaptive capacity at younger ages and adaptive capacity at old ages). But generally, it confirms our earlier observations that there is no universal behavior of the decline in adaptive capacity for different physiological variables in females and males (4, 26).

Figure 2 displays box plots of parameter estimates in different variants of *D*_M_ applied to female and male data. This figure illustrates the sex differences in the components of the model described above from the perspective of model’s parameters. It also shows the estimates of parameters σ_0_ and σ_1_ indicating that females and males also differ in terms of variability of *D*_M_ (both at baseline and dynamic variability over age), and, respectively, in terms of variability of the underlying physiological variables, with females having higher variability than males.

### Advantages, Limitations, and Further Perspectives

The advantages of the presented mathematical model of age trajectories of physiological dysregulation, aging, and mortality combine those of the underlying model (SPM) and the measure of physiological dysregulation (*D*_M_), i.e., a robust measure of physiological dysregulation with a biologically explicit and easily interpretable model of age-related changes in physiology in relation to mortality (4, 5, 8, 10, 26, 27). An additional advantage is that the use of this measure of physiological dysregulation (*D*_M_) allows us to perform analysis of a one-dimensional SPM instead of using its multidimensional version.

Physiological variables can be analyzed within the SPM approach by using them explicitly in the hazard or in the summary measures such as the distance *D*_M_. The first approach is more flexible in the sense that we have the capability to specify parameters of the model for each variable separately and, thus, to investigate their effects on the time-to-event outcome in detail, as well as to make inference on their dynamic properties from respective parameters in Eq. 3. However, this flexibility has its price as we deal with a multidimensional model and the computational workload in the likelihood estimation procedure is essential. Therefore, if one is interested in the combined effect of the variables on the outcome of interest (and also is not investigating the dynamic properties of each individual variable) then the approach implementing the summary measures such as *D*_M_ becomes beneficial. It substantially reduces the computational burden as it works in a one-dimensional setting while it still has the same components, which can be interpreted in the context of aging-related processes. In the case of a large number of physiological variables this may result in a considerable computational advantage because the calculations of *D*_M_ are much faster than the maximization of the likelihood function of the multidimensional SPM for the original set of physiological variables.

From a theoretical standpoint, use of a global measure of physiological dysregulation is likely to be more robust and less noisy than individual biomarkers, which may vary for many reasons unrelated to long-term aging processes. The interpretation of *D*_M_ as a robust measure is enhanced by the fact that our results here are highly concordant with previous studies on the Women’s Health and Aging Study (WHAS), the Baltimore Longitudinal Study on Aging (BLSA), and InCHIANTI (5, 20, 44), despite major differences in (a) statistical approach; (b) biomarker choice (only three of the biomarkers here – CH, glucose, and hematocrit – were among the 40+ used in those studies); (c) participant characteristics (e.g., WHAS contained exclusively older women from the Baltimore area; InCHIANTI was in Italy); and (d) follow-up time (~5–10 years for WHAS, BLSA, and InCHIANTI versus ~55 years here). This replication of results confirms the idea that *D*_M_ represents an underlying system-level property of physiological dysregulation that can be detected and interpreted relatively robustly under different contexts and with different markers.

The proposed model provides an approach to measure the impact of systemic dysregulation in an organism on mortality risk, which is an alternative to that using a cumulative measure of health deterioration such as an index of cumulative deficits or deficits index (DI) (45, 46), also an efficient approach to investigate aging-related processes of health deterioration. The DI, which takes into account the cumulative contribution of different variables (including those with possibly minor effects of individual variables on the risk) on mortality risk, can also be implemented in the SPM as in our earlier work (22). Remarkably, both approaches revealed similar sex-specific differences in dynamics of model’s components, see Figure 1 here and Figure 1 in Yashin et al. (22): baseline mortality is higher in males; U-shape (J-shape) of mortality as a function of DI (*D*_M_) is narrower and it narrows faster with age in males; and mean allostatic trajectories are higher and increase faster in females (with respective trajectories estimated at zero for males in the case of DI). Such similarities are observed despite, again, major differences in (a) the measure used in the model (*D*_M_ and DI); (b) variables used in construction of respective measures (biomarkers here versus questions from the survey questionnaires in the DI studies); (c) participant characteristics, sample size, and follow-up time (the model with DI was applied to the National Long Term Care Survey (NLTCS) data, a nationally representative survey of more than 49,000 Medicare enrollees); and (d) specifications of the SPM [gamma-Gompertz (logistic) baseline hazards, non-symmetric U-shapes, non-zero “norms,” quadratic functions for mean allostatic trajectories, and constant feedback coefficients in the SPM applied to the NLTCS data]. This similarity in the results obtained using these two different approaches can thus indicate manifestation of different aspects of the same general process of aging-related deterioration in an organism that cause sexual dimorphism in the dynamics of aging-related processes.

There are two limitations in the implementation of the model described in this paper. First, we note that, as a single imputation method was used, the uncertainty and SEs could in general be underestimated. Another limitation is that it defines the “normal” state of physiological variables from which deviations are evaluated by *D*_M_ using the mean values at the baseline exam in individuals 40 years and younger. That is, it is assumed that these values minimize the risk of death (as a function of these physiological variables) at all ages. Note that, if we insert the formula of *D*_M_ (Eq. 1) in Eq. 4 then, essentially, the vector of means $\bar{X}$ in *D*_M_ represents the “physiological norm” or “optimum” for respective physiological variables in the corresponding multidimensional SPM for the original variables included in the definition of *D*_M_. Thus, we actually assume here that this “physiological optimum” is age independent. While this assumption can be true for some variables, for other variables it is likely to be incorrect. Many hormone-related variables experience changes in accordance with the ontogenetic program, and these changes are likely to modify the optimal value for these variables [e.g., this could be manifested by the menopause; see also discussion on age-dependent physiological norms in Yashin et al. (3)]. Thus, one possibility to enhance the results presented here is to assume age-dependent “optima” in the definition of *D*_M_. However, this can generally be a challenging problem because one needs to decide which values to use to define the “optima” for different ages before performing analyses of SPM rather than estimate these “physiological optima” from the SPM itself as in our earlier applications (3, 4, 21–24). A possible remedy could be to use a “surrogate” definition of the “physiological optimum” suggested in Yashin et al. (26) taking the average age trajectories of physiological variables for long-lived individuals (say, 90+) on the premise that the long-lived individuals are those who, on the average, managed to keep the age trajectories of physiological variables close to the optimal ones that minimize the risk of death at respective ages. However, there are still computational challenges in this approach as one will need a large enough number of long-lived individuals to reliably estimate the means and the variance–covariance matrices in respective age groups as needed for *D*_M_. Alternatively, we can relax the assumption on zero “optimum” *f*_0_(*t*) (and this is also a testable hypothesis in this approach). Although in the current implementation of the model a non-zero “optimum” for *D*_M_ could mean different things depending on the patterns of changes in the underlying physiological variables, this approach could provide insights on whether the values of biomarkers that are “optimal” for younger individuals are still “optimal” at older ages.

Another direction for applications of the approach presented in this paper is to investigate genetic effects of different candidate genes or single-nucleotide polymorphisms (SNPs) on the measures of physiological dysregulation and on respective aging-related characteristics such as mean allostatic trajectory, resistance to stresses or adaptive capacity which can be evaluated in SPM. For example, one can evaluate carriers of which alleles or genotypes have higher levels of physiological dysregulation, a faster increase in systemic dysregulation with age, a faster decline in resistance to stresses or adaptive capacity calculating respective estimates of SPM components for carriers of different alleles or genotypes. This can be done using either a stratified analysis by genotype or allele using the original SPM (8) as we did here stratifying by sex or implementing *D*_M_ in the genetic SPM (9), which has an advantage of using additional information on non-genotyped individuals to increase the power. This can also be done either for individual genes/SNPs or for cumulative “genetic doses,” that is the variable calculating the number of some alleles in an individual’s genome that were pre-selected using some criteria or analyses [e.g., “longevity alleles” as in Yashin et al. (27)].

The desire to have useful biological interpretation of the results of analyses stimulates the need for further development of concepts and models describing the aging-related changes developing in the human body. Although the “dysregulation” variable (*D*_M_) is useful because it allows us to quantify aging-related changes showing how the particular individual differs from the “ideal” standard (e.g., observed in healthy young adults), its applications to studying mechanisms of aging-related changes requires its further development. From the point of view of this paper, the dysregulation is the result of everything that causes the constructed measure (*D*_M_) to deviate from zero where the zero value characterizes the healthy young adults. At the same time, such measure could be too general if one would like to understand causes and mechanisms of aging-related changes, for example, to produce useful recommendations for improving person’s health. One approach might be to examine *D*_M_ by specific physiological system (47). Alternatively, in Ukraintseva and Yashin (48) and Arbeev et al. (49), the importance of distinguishing among three components of aging-related changes was emphasized. These include basal (senescent), ontogenetic, and exposure related components. The approach described above has a potential to include these components into the model and use them to address questions about various mechanisms driving dynamics of aging-related changes during the life course. For example, allostatic load can be linked with the exposure-related component. Changes in physiological norm can be linked with the ontogenetic component and changes in stress resistance and adaptive capacity with the basal (senescent) component. All three components contribute to dysregulation in physiological state, although their interaction may sometimes have side effects that might slow down or even reduce *D*_M_. For example, the reduction of adaptive capacity may slow down accumulation of *D*_M_ due to weakening negative feedback, especially when *f*_1_(*t*) continues to increase. This process could partly be due to compensatory adaptation. The effects, however, may not last long because in case of weak feedback regulation the “noise” component in Eq. 3 will make stronger effects on *Y*(*t*). Note that different components of aging-related changes can be evaluated from available longitudinal data (24, 27). More detailed analyses of dysregulation components will be performed elsewhere.

## Conclusion

Implementation of the measure of physiological dysregulation (Mahalanobis distance) in the framework of the SPM of aging allows one to investigate physiological dysregulation in relation to different hidden mechanisms of aging-related changes, and, ultimately, to yield estimates of how such dynamic relationships can produce an increase in the risk of death with age and how it may be related to the observed sex-specific differences in mortality risks. Results of application of the method to individuals from the Framingham original cohort indicate that physiological dysregulation increases with age; that increased dysregulation results in increased mortality, and increasingly so with age; and that, in most but not all cases, there is a decreasing ability to return to baseline physiological state with age. We also show substantial sex differences in these processes, with women becoming dysregulated more quickly but with men showing a much greater sensitivity to dysregulation in terms of mortality risk.

## Author Contributions

Designed the study: KA and AC; performed statistical analyses and data preparation: KA and LA; contributed software for computation of statistical distance: AC and EM; contributed to methodological work with stochastic process model implementing statistical distance: KA, ES, IA, and AY; wrote the paper: KA, AC, and EM; contributed to the final version and interpretation of results: ES, AK, SU, KC, and AY.

## Conflict of Interest Statement

The authors declare that the research was conducted in the absence of any commercial or financial relationships that could be construed as a potential conflict of interest.
