# Supplementary material for: Optimal Versus Realized Trajectories of Physiological Dysregulation in Aging and Their Relation to Sex-Specific Mortality Risk
Source: Front Public Health. 2016 Jan 25;4:3. doi: 10.3389/fpubh.2016.00003 (PMC4725219; doi:10.3389/fpubh.2016.00003)
Supplement: Supplementary file 1 [file Table_1.DOCX]

Supplementary Material

Optimal versus Realized Trajectories of Physiological Dysregulation in Aging and their Relation to Sex-Specific Mortality Risk

Konstantin G. Arbeev^*^, Alan A. Cohen, Liubov S. Arbeeva, Emmanuel Milot, Eric Stallard, Alexander M. Kulminski, Igor Akushevich, Svetlana V. Ukraintseva, Kaare Christensen, Anatoliy I. Yashin

*** Correspondence:** Konstantin G. Arbeev: [ka29@duke.edu](mailto:ka29@duke.edu)

# Supplementary Tables

**Supplementary Table 1:** Likelihood ratio test statistics for different restricted models (with restrictions on the parameters specified in column **Restricted Model**)

| **Sex** | ***D*_M_ Variables** | **Restricted Model** | **P-value** | **Statistics** | **df** |
| --- | --- | --- | --- | --- | --- |
| females | BG, BMI, CH, DBP, HC, SBP, VR | *a*(*t*)=const | <0.0001 | 19.36 | 1 |
| females | BG, BMI, CH, DBP, HC, SBP, VR | *f*_1_(*t*)=const | <0.0001 | 2343.79 | 1 |
| females | BG, BMI, CH, DBP, HC, SBP, VR | *f*_1_(*t*)=0 | <0.0001 | 10956.60 | 2 |
| females | BG, BMI, CH, DBP, HC, SBP, VR | *Q*(*t*)=const | 0.0046 | 8.02 | 1 |
| females | BG, BMI, CH, DBP, HC, SBP, VR | *Q*(*t*)=0 | 0.0001 | 17.94 | 2 |
| females | BG, BMI, CH, DBP, HC, PP, VR | *a*(*t*)=const | <0.0001 | 15.66 | 1 |
| females | BG, BMI, CH, DBP, HC, PP, VR | *f*_1_(*t*)=const | <0.0001 | 1966.57 | 1 |
| females | BG, BMI, CH, DBP, HC, PP, VR | *f*_1_(*t*)=0 | <0.0001 | 11103.29 | 2 |
| females | BG, BMI, CH, DBP, HC, PP, VR | *Q*(*t*)=const | 0.0054 | 7.73 | 1 |
| females | BG, BMI, CH, DBP, HC, PP, VR | *Q*(*t*)=0 | 0.0002 | 16.69 | 2 |
| females | BG, BMI, CH, HC, PP, SBP, VR | *a*(*t*)=const | <0.0001 | 27.84 | 1 |
| females | BG, BMI, CH, HC, PP, SBP, VR | *f*_1_(*t*)=const | <0.0001 | 1837.79 | 1 |
| females | BG, BMI, CH, HC, PP, SBP, VR | *f*_1_(*t*)=0 | <0.0001 | 11427.22 | 2 |
| females | BG, BMI, CH, HC, PP, SBP, VR | *Q*(*t*)=const | 0.0054 | 7.73 | 1 |
| females | BG, BMI, CH, HC, PP, SBP, VR | *Q*(*t*)=0 | 0.0003 | 16.54 | 2 |
| females | BG, BMI, CH, DBP, SBP | *a*(*t*)=const | <0.0001 | 37.20 | 1 |
| females | BG, BMI, CH, DBP, SBP | *f*_1_(*t*)=const | <0.0001 | 2886.90 | 1 |
| females | BG, BMI, CH, DBP, SBP | *f*_1_(*t*)=0 | <0.0001 | 10909.27 | 2 |
| females | BG, BMI, CH, DBP, SBP | *Q*(*t*)=const | 0.0377 | 4.32 | 1 |
| females | BG, BMI, CH, DBP, SBP | *Q*(*t*)=0 | <0.0001 | 27.28 | 2 |
| females | BG, BMI, CH, DBP, PP | *a*(*t*)=const | <0.0001 | 50.62 | 1 |
| females | BG, BMI, CH, DBP, PP | *f*_1_(*t*)=const | <0.0001 | 2428.78 | 1 |
| females | BG, BMI, CH, DBP, PP | *f*_1_(*t*)=0 | <0.0001 | 11240.57 | 2 |
| females | BG, BMI, CH, DBP, PP | *Q*(*t*)=const | 0.0099 | 6.64 | 1 |
| females | BG, BMI, CH, DBP, PP | *Q*(*t*)=0 | <0.0001 | 28.28 | 2 |
| females | BG, BMI, CH, PP, SBP | *a*(*t*)=const | <0.0001 | 66.63 | 1 |
| females | BG, BMI, CH, PP, SBP | *f*_1_(*t*)=const | <0.0001 | 2183.90 | 1 |
| females | BG, BMI, CH, PP, SBP | *f*_1_(*t*)=0 | <0.0001 | 11755.83 | 2 |
| females | BG, BMI, CH, PP, SBP | *Q*(*t*)=const | 0.0067 | 7.36 | 1 |
| females | BG, BMI, CH, PP, SBP | *Q*(*t*)=0 | <0.0001 | 27.86 | 2 |
| females | BMI, CH, DBP, HC, SBP, VR | *a*(*t*)=const | 0.0803 | 3.06 | 1 |
| females | BMI, CH, DBP, HC, SBP, VR | *f*_1_(*t*)=const | <0.0001 | 2383.06 | 1 |
| females | BMI, CH, DBP, HC, SBP, VR | *f*_1_(*t*)=0 | <0.0001 | 10664.10 | 2 |
| females | BMI, CH, DBP, HC, SBP, VR | *Q*(*t*)=const | 0.0061 | 7.51 | 1 |
| females | BMI, CH, DBP, HC, SBP, VR | *Q*(*t*)=0 | <0.0001 | 30.50 | 2 |
| females | BMI, CH, DBP, HC, PP, VR | *a*(*t*)=const | 0.2823 | 1.16 | 1 |
| females | BMI, CH, DBP, HC, PP, VR | *f*_1_(*t*)=const | <0.0001 | 1928.15 | 1 |
| females | BMI, CH, DBP, HC, PP, VR | *f*_1_(*t*)=0 | <0.0001 | 10807.02 | 2 |
| females | BMI, CH, DBP, HC, PP, VR | *Q*(*t*)=const | 0.0032 | 8.68 | 1 |
| females | BMI, CH, DBP, HC, PP, VR | *Q*(*t*)=0 | <0.0001 | 30.82 | 2 |
| females | BMI, CH, HC, PP, SBP, VR | *a*(*t*)=const | 0.0127 | 6.21 | 1 |
| females | BMI, CH, HC, PP, SBP, VR | *f*_1_(*t*)=const | <0.0001 | 1791.24 | 1 |
| females | BMI, CH, HC, PP, SBP, VR | *f*_1_(*t*)=0 | <0.0001 | 11129.97 | 2 |
| females | BMI, CH, HC, PP, SBP, VR | *Q*(*t*)=const | 0.0031 | 8.72 | 1 |
| females | BMI, CH, HC, PP, SBP, VR | *Q*(*t*)=0 | <0.0001 | 29.58 | 2 |
| females | BG, CH, DBP, HC, SBP, VR | *a*(*t*)=const | <0.0001 | 41.27 | 1 |
| females | BG, CH, DBP, HC, SBP, VR | *f*_1_(*t*)=const | <0.0001 | 2450.33 | 1 |
| females | BG, CH, DBP, HC, SBP, VR | *f*_1_(*t*)=0 | <0.0001 | 11058.92 | 2 |
| females | BG, CH, DBP, HC, SBP, VR | *Q*(*t*)=const | 0.0017 | 9.82 | 1 |
| females | BG, CH, DBP, HC, SBP, VR | *Q*(*t*)=0 | <0.0001 | 41.85 | 2 |
| females | BG, CH, DBP, HC, PP, VR | *a*(*t*)=const | <0.0001 | 33.66 | 1 |
| females | BG, CH, DBP, HC, PP, VR | *f*_1_(*t*)=const | <0.0001 | 2055.27 | 1 |
| females | BG, CH, DBP, HC, PP, VR | *f*_1_(*t*)=0 | <0.0001 | 11247.50 | 2 |
| females | BG, CH, DBP, HC, PP, VR | *Q*(*t*)=const | 0.0006 | 11.67 | 1 |
| females | BG, CH, DBP, HC, PP, VR | *Q*(*t*)=0 | <0.0001 | 41.08 | 2 |
| females | BG, CH, HC, PP, SBP, VR | *a*(*t*)=const | <0.0001 | 49.60 | 1 |
| females | BG, CH, HC, PP, SBP, VR | *f*_1_(*t*)=const | <0.0001 | 1956.24 | 1 |
| females | BG, CH, HC, PP, SBP, VR | *f*_1_(*t*)=0 | <0.0001 | 11589.99 | 2 |
| females | BG, CH, HC, PP, SBP, VR | *Q*(*t*)=const | 0.0005 | 12.08 | 1 |
| females | BG, CH, HC, PP, SBP, VR | *Q*(*t*)=0 | <0.0001 | 41.50 | 2 |
| females | BG, BMI, DBP, HC, SBP, VR | *a*(*t*)=const | 0.0032 | 8.69 | 1 |
| females | BG, BMI, DBP, HC, SBP, VR | *f*_1_(*t*)=const | <0.0001 | 2535.76 | 1 |
| females | BG, BMI, DBP, HC, SBP, VR | *f*_1_(*t*)=0 | <0.0001 | 10680.65 | 2 |
| females | BG, BMI, DBP, HC, SBP, VR | *Q*(*t*)=const | 0.0009 | 11.04 | 1 |
| females | BG, BMI, DBP, HC, SBP, VR | *Q*(*t*)=0 | <0.0001 | 19.94 | 2 |
| females | BG, BMI, DBP, HC, PP, VR | *a*(*t*)=const | 0.0186 | 5.54 | 1 |
| females | BG, BMI, DBP, HC, PP, VR | *f*_1_(*t*)=const | <0.0001 | 2199.88 | 1 |
| females | BG, BMI, DBP, HC, PP, VR | *f*_1_(*t*)=0 | <0.0001 | 10928.72 | 2 |
| females | BG, BMI, DBP, HC, PP, VR | *Q*(*t*)=const | 0.0018 | 9.70 | 1 |
| females | BG, BMI, DBP, HC, PP, VR | *Q*(*t*)=0 | 0.0002 | 16.93 | 2 |
| females | BG, BMI, HC, PP, SBP, VR | *a*(*t*)=const | 0.0001 | 14.45 | 1 |
| females | BG, BMI, HC, PP, SBP, VR | *f*_1_(*t*)=const | <0.0001 | 2061.67 | 1 |
| females | BG, BMI, HC, PP, SBP, VR | *f*_1_(*t*)=0 | <0.0001 | 11281.88 | 2 |
| females | BG, BMI, HC, PP, SBP, VR | *Q*(*t*)=const | 0.0025 | 9.13 | 1 |
| females | BG, BMI, HC, PP, SBP, VR | *Q*(*t*)=0 | 0.0004 | 15.78 | 2 |
| females | BG, BMI, CH, HC, SBP, VR | *a*(*t*)=const | 0.2965 | 1.09 | 1 |
| females | BG, BMI, CH, HC, SBP, VR | *f*_1_(*t*)=const | <0.0001 | 1213.08 | 1 |
| females | BG, BMI, CH, HC, SBP, VR | *f*_1_(*t*)=0 | <0.0001 | 10604.59 | 2 |
| females | BG, BMI, CH, HC, SBP, VR | *Q*(*t*)=const | 0.0101 | 6.62 | 1 |
| females | BG, BMI, CH, HC, SBP, VR | *Q*(*t*)=0 | 0.0007 | 14.59 | 2 |
| females | BG, BMI, CH, HC, PP, VR | *a*(*t*)=const | <0.0001 | 21.92 | 1 |
| females | BG, BMI, CH, HC, PP, VR | *f*_1_(*t*)=const | <0.0001 | 2029.09 | 1 |
| females | BG, BMI, CH, HC, PP, VR | *f*_1_(*t*)=0 | <0.0001 | 10984.37 | 2 |
| females | BG, BMI, CH, HC, PP, VR | *Q*(*t*)=const | 0.0067 | 7.36 | 1 |
| females | BG, BMI, CH, HC, PP, VR | *Q*(*t*)=0 | 0.0002 | 16.82 | 2 |
| females | BG, BMI, CH, DBP, SBP, VR | *a*(*t*)=const | <0.0001 | 31.98 | 1 |
| females | BG, BMI, CH, DBP, SBP, VR | *f*_1_(*t*)=const | <0.0001 | 2965.17 | 1 |
| females | BG, BMI, CH, DBP, SBP, VR | *f*_1_(*t*)=0 | <0.0001 | 10962.63 | 2 |
| females | BG, BMI, CH, DBP, SBP, VR | *Q*(*t*)=const | 0.0023 | 9.33 | 1 |
| females | BG, BMI, CH, DBP, SBP, VR | *Q*(*t*)=0 | <0.0001 | 24.19 | 2 |
| females | BG, BMI, CH, DBP, PP, VR | *a*(*t*)=const | <0.0001 | 30.92 | 1 |
| females | BG, BMI, CH, DBP, PP, VR | *f*_1_(*t*)=const | <0.0001 | 2538.32 | 1 |
| females | BG, BMI, CH, DBP, PP, VR | *f*_1_(*t*)=0 | <0.0001 | 11349.63 | 2 |
| females | BG, BMI, CH, DBP, PP, VR | *Q*(*t*)=const | 0.0008 | 11.15 | 1 |
| females | BG, BMI, CH, DBP, PP, VR | *Q*(*t*)=0 | <0.0001 | 24.81 | 2 |
| females | BG, BMI, CH, PP, SBP, VR | *a*(*t*)=const | <0.0001 | 34.05 | 1 |
| females | BG, BMI, CH, PP, SBP, VR | *f*_1_(*t*)=const | <0.0001 | 2374.27 | 1 |
| females | BG, BMI, CH, PP, SBP, VR | *f*_1_(*t*)=0 | <0.0001 | 11845.06 | 2 |
| females | BG, BMI, CH, PP, SBP, VR | *Q*(*t*)=const | 0.0009 | 10.93 | 1 |
| females | BG, BMI, CH, PP, SBP, VR | *Q*(*t*)=0 | <0.0001 | 23.32 | 2 |
| females | BG, BMI, CH, DBP, HC, VR | *a*(*t*)=const | 1 | 0.00 | 1 |
| females | BG, BMI, CH, DBP, HC, VR | *f*_1_(*t*)=const | <0.0001 | 688.45 | 1 |
| females | BG, BMI, CH, DBP, HC, VR | *f*_1_(*t*)=0 | <0.0001 | 10842.42 | 2 |
| females | BG, BMI, CH, DBP, HC, VR | *Q*(*t*)=const | 0.0045 | 8.06 | 1 |
| females | BG, BMI, CH, DBP, HC, VR | *Q*(*t*)=0 | 0.0002 | 17.12 | 2 |
| females | BG, BMI, CH, DBP, HC, SBP | *a*(*t*)=const | <0.0001 | 23.80 | 1 |
| females | BG, BMI, CH, DBP, HC, SBP | *f*_1_(*t*)=const | <0.0001 | 2219.37 | 1 |
| females | BG, BMI, CH, DBP, HC, SBP | *f*_1_(*t*)=0 | <0.0001 | 10601.85 | 2 |
| females | BG, BMI, CH, DBP, HC, SBP | *Q*(*t*)=const | 0.0063 | 7.47 | 1 |
| females | BG, BMI, CH, DBP, HC, SBP | *Q*(*t*)=0 | 0.0002 | 17.33 | 2 |
| females | BG, BMI, CH, DBP, HC, PP | *a*(*t*)=const | <0.0001 | 24.97 | 1 |
| females | BG, BMI, CH, DBP, HC, PP | *f*_1_(*t*)=const | <0.0001 | 1831.97 | 1 |
| females | BG, BMI, CH, DBP, HC, PP | *f*_1_(*t*)=0 | <0.0001 | 10707.85 | 2 |
| females | BG, BMI, CH, DBP, HC, PP | *Q*(*t*)=const | 0.0074 | 7.17 | 1 |
| females | BG, BMI, CH, DBP, HC, PP | *Q*(*t*)=0 | 0.0003 | 15.99 | 2 |
| females | BG, BMI, CH, HC, PP, SBP | *a*(*t*)=const | <0.0001 | 44.25 | 1 |
| females | BG, BMI, CH, HC, PP, SBP | *f*_1_(*t*)=const | <0.0001 | 1665.08 | 1 |
| females | BG, BMI, CH, HC, PP, SBP | *f*_1_(*t*)=0 | <0.0001 | 11032.98 | 2 |
| females | BG, BMI, CH, HC, PP, SBP | *Q*(*t*)=const | 0.0069 | 7.31 | 1 |
| females | BG, BMI, CH, HC, PP, SBP | *Q*(*t*)=0 | 0.0003 | 16.02 | 2 |
| males | BG, BMI, CH, DBP, HC, SBP, VR | *a*(*t*)=const | 0.7592 | 0.09 | 1 |
| males | BG, BMI, CH, DBP, HC, SBP, VR | *f*_1_(*t*)=const | <0.0001 | 872.23 | 1 |
| males | BG, BMI, CH, DBP, HC, SBP, VR | *f*_1_(*t*)=0 | <0.0001 | 8880.01 | 2 |
| males | BG, BMI, CH, DBP, HC, SBP, VR | *Q*(*t*)=const | <0.0001 | 19.06 | 1 |
| males | BG, BMI, CH, DBP, HC, SBP, VR | *Q*(*t*)=0 | <0.0001 | 46.62 | 2 |
| males | BG, BMI, CH, DBP, HC, PP, VR | *a*(*t*)=const | 0.2432 | 1.36 | 1 |
| males | BG, BMI, CH, DBP, HC, PP, VR | *f*_1_(*t*)=const | <0.0001 | 735.12 | 1 |
| males | BG, BMI, CH, DBP, HC, PP, VR | *f*_1_(*t*)=0 | <0.0001 | 9208.64 | 2 |
| males | BG, BMI, CH, DBP, HC, PP, VR | *Q*(*t*)=const | <0.0001 | 19.10 | 1 |
| males | BG, BMI, CH, DBP, HC, PP, VR | *Q*(*t*)=0 | <0.0001 | 46.50 | 2 |
| males | BG, BMI, CH, HC, PP, SBP, VR | *a*(*t*)=const | 0.0185 | 5.54 | 1 |
| males | BG, BMI, CH, HC, PP, SBP, VR | *f*_1_(*t*)=const | <0.0001 | 646.50 | 1 |
| males | BG, BMI, CH, HC, PP, SBP, VR | *f*_1_(*t*)=0 | <0.0001 | 9558.48 | 2 |
| males | BG, BMI, CH, HC, PP, SBP, VR | *Q*(*t*)=const | <0.0001 | 18.02 | 1 |
| males | BG, BMI, CH, HC, PP, SBP, VR | *Q*(*t*)=0 | <0.0001 | 40.36 | 2 |
| males | BG, BMI, CH, DBP, SBP | *a*(*t*)=const | <0.0001 | 77.10 | 1 |
| males | BG, BMI, CH, DBP, SBP | *f*_1_(*t*)=const | <0.0001 | 926.18 | 1 |
| males | BG, BMI, CH, DBP, SBP | *f*_1_(*t*)=0 | <0.0001 | 8480.75 | 2 |
| males | BG, BMI, CH, DBP, SBP | *Q*(*t*)=const | <0.0001 | 16.62 | 1 |
| males | BG, BMI, CH, DBP, SBP | *Q*(*t*)=0 | <0.0001 | 69.25 | 2 |
| males | BG, BMI, CH, DBP, PP | *a*(*t*)=const | <0.0001 | 99.57 | 1 |
| males | BG, BMI, CH, DBP, PP | *f*_1_(*t*)=const | <0.0001 | 782.81 | 1 |
| males | BG, BMI, CH, DBP, PP | *f*_1_(*t*)=0 | <0.0001 | 9047.09 | 2 |
| males | BG, BMI, CH, DBP, PP | *Q*(*t*)=const | <0.0001 | 19.57 | 1 |
| males | BG, BMI, CH, DBP, PP | *Q*(*t*)=0 | <0.0001 | 71.20 | 2 |
| males | BG, BMI, CH, PP, SBP | *a*(*t*)=const | <0.0001 | 107.76 | 1 |
| males | BG, BMI, CH, PP, SBP | *f*_1_(*t*)=const | <0.0001 | 647.60 | 1 |
| males | BG, BMI, CH, PP, SBP | *f*_1_(*t*)=0 | <0.0001 | 9625.76 | 2 |
| males | BG, BMI, CH, PP, SBP | *Q*(*t*)=const | <0.0001 | 19.66 | 1 |
| males | BG, BMI, CH, PP, SBP | *Q*(*t*)=0 | <0.0001 | 61.49 | 2 |
| males | BMI, CH, DBP, HC, SBP, VR | *a*(*t*)=const | 1 | 0.00 | 1 |
| males | BMI, CH, DBP, HC, SBP, VR | *f*_1_(*t*)=const | <0.0001 | 855.00 | 1 |
| males | BMI, CH, DBP, HC, SBP, VR | *f*_1_(*t*)=0 | <0.0001 | 8561.50 | 2 |
| males | BMI, CH, DBP, HC, SBP, VR | *Q*(*t*)=const | <0.0001 | 23.97 | 1 |
| males | BMI, CH, DBP, HC, SBP, VR | *Q*(*t*)=0 | <0.0001 | 67.71 | 2 |
| males | BMI, CH, DBP, HC, PP, VR | *a*(*t*)=const | 1 | 0.00 | 1 |
| males | BMI, CH, DBP, HC, PP, VR | *f*_1_(*t*)=const | <0.0001 | 693.33 | 1 |
| males | BMI, CH, DBP, HC, PP, VR | *f*_1_(*t*)=0 | <0.0001 | 8918.79 | 2 |
| males | BMI, CH, DBP, HC, PP, VR | *Q*(*t*)=const | <0.0001 | 24.26 | 1 |
| males | BMI, CH, DBP, HC, PP, VR | *Q*(*t*)=0 | <0.0001 | 66.72 | 2 |
| males | BMI, CH, HC, PP, SBP, VR | *a*(*t*)=const | 1 | 0.00 | 1 |
| males | BMI, CH, HC, PP, SBP, VR | *f*_1_(*t*)=const | <0.0001 | 605.51 | 1 |
| males | BMI, CH, HC, PP, SBP, VR | *f*_1_(*t*)=0 | <0.0001 | 9299.65 | 2 |
| males | BMI, CH, HC, PP, SBP, VR | *Q*(*t*)=const | <0.0001 | 23.32 | 1 |
| males | BMI, CH, HC, PP, SBP, VR | *Q*(*t*)=0 | <0.0001 | 60.07 | 2 |
| males | BG, CH, DBP, HC, SBP, VR | *a*(*t*)=const | 0.3802 | 0.77 | 1 |
| males | BG, CH, DBP, HC, SBP, VR | *f*_1_(*t*)=const | <0.0001 | 918.91 | 1 |
| males | BG, CH, DBP, HC, SBP, VR | *f*_1_(*t*)=0 | <0.0001 | 8939.24 | 2 |
| males | BG, CH, DBP, HC, SBP, VR | *Q*(*t*)=const | <0.0001 | 25.54 | 1 |
| males | BG, CH, DBP, HC, SBP, VR | *Q*(*t*)=0 | <0.0001 | 67.73 | 2 |
| males | BG, CH, DBP, HC, PP, VR | *a*(*t*)=const | 0.1443 | 2.13 | 1 |
| males | BG, CH, DBP, HC, PP, VR | *f*_1_(*t*)=const | <0.0001 | 780.12 | 1 |
| males | BG, CH, DBP, HC, PP, VR | *f*_1_(*t*)=0 | <0.0001 | 9351.96 | 2 |
| males | BG, CH, DBP, HC, PP, VR | *Q*(*t*)=const | <0.0001 | 27.33 | 1 |
| males | BG, CH, DBP, HC, PP, VR | *Q*(*t*)=0 | <0.0001 | 70.30 | 2 |
| males | BG, CH, HC, PP, SBP, VR | *a*(*t*)=const | 0.0233 | 5.15 | 1 |
| males | BG, CH, HC, PP, SBP, VR | *f*_1_(*t*)=const | <0.0001 | 700.07 | 1 |
| males | BG, CH, HC, PP, SBP, VR | *f*_1_(*t*)=0 | <0.0001 | 9713.40 | 2 |
| males | BG, CH, HC, PP, SBP, VR | *Q*(*t*)=const | <0.0001 | 26.16 | 1 |
| males | BG, CH, HC, PP, SBP, VR | *Q*(*t*)=0 | <0.0001 | 63.15 | 2 |
| males | BG, BMI, DBP, HC, SBP, VR | *a*(*t*)=const | 0.3232 | 0.98 | 1 |
| males | BG, BMI, DBP, HC, SBP, VR | *f*_1_(*t*)=const | <0.0001 | 1121.90 | 1 |
| males | BG, BMI, DBP, HC, SBP, VR | *f*_1_(*t*)=0 | <0.0001 | 8861.58 | 2 |
| males | BG, BMI, DBP, HC, SBP, VR | *Q*(*t*)=const | <0.0001 | 23.16 | 1 |
| males | BG, BMI, DBP, HC, SBP, VR | *Q*(*t*)=0 | <0.0001 | 48.30 | 2 |
| males | BG, BMI, DBP, HC, PP, VR | *a*(*t*)=const | 0.3163 | 1.00 | 1 |
| males | BG, BMI, DBP, HC, PP, VR | *f*_1_(*t*)=const | <0.0001 | 987.26 | 1 |
| males | BG, BMI, DBP, HC, PP, VR | *f*_1_(*t*)=0 | <0.0001 | 9282.04 | 2 |
| males | BG, BMI, DBP, HC, PP, VR | *Q*(*t*)=const | <0.0001 | 22.42 | 1 |
| males | BG, BMI, DBP, HC, PP, VR | *Q*(*t*)=0 | <0.0001 | 46.56 | 2 |
| males | BG, BMI, HC, PP, SBP, VR | *a*(*t*)=const | 0.0802 | 3.06 | 1 |
| males | BG, BMI, HC, PP, SBP, VR | *f*_1_(*t*)=const | <0.0001 | 894.13 | 1 |
| males | BG, BMI, HC, PP, SBP, VR | *f*_1_(*t*)=0 | <0.0001 | 9696.58 | 2 |
| males | BG, BMI, HC, PP, SBP, VR | *Q*(*t*)=const | <0.0001 | 20.15 | 1 |
| males | BG, BMI, HC, PP, SBP, VR | *Q*(*t*)=0 | <0.0001 | 39.00 | 2 |
| males | BG, BMI, CH, HC, SBP, VR | *a*(*t*)=const | 1 | 0.00 | 1 |
| males | BG, BMI, CH, HC, SBP, VR | *f*_1_(*t*)=const | <0.0001 | 419.43 | 1 |
| males | BG, BMI, CH, HC, SBP, VR | *f*_1_(*t*)=0 | <0.0001 | 8816.71 | 2 |
| males | BG, BMI, CH, HC, SBP, VR | *Q*(*t*)=const | <0.0001 | 16.89 | 1 |
| males | BG, BMI, CH, HC, SBP, VR | *Q*(*t*)=0 | <0.0001 | 40.69 | 2 |
| males | BG, BMI, CH, HC, PP, VR | *a*(*t*)=const | 0.0249 | 5.03 | 1 |
| males | BG, BMI, CH, HC, PP, VR | *f*_1_(*t*)=const | <0.0001 | 633.31 | 1 |
| males | BG, BMI, CH, HC, PP, VR | *f*_1_(*t*)=0 | <0.0001 | 8962.67 | 2 |
| males | BG, BMI, CH, HC, PP, VR | *Q*(*t*)=const | 0.0001 | 15.00 | 1 |
| males | BG, BMI, CH, HC, PP, VR | *Q*(*t*)=0 | <0.0001 | 34.49 | 2 |
| males | BG, BMI, CH, DBP, SBP, VR | *a*(*t*)=const | <0.0001 | 33.01 | 1 |
| males | BG, BMI, CH, DBP, SBP, VR | *f*_1_(*t*)=const | <0.0001 | 1073.41 | 1 |
| males | BG, BMI, CH, DBP, SBP, VR | *f*_1_(*t*)=0 | <0.0001 | 8697.15 | 2 |
| males | BG, BMI, CH, DBP, SBP, VR | *Q*(*t*)=const | <0.0001 | 24.90 | 1 |
| males | BG, BMI, CH, DBP, SBP, VR | *Q*(*t*)=0 | <0.0001 | 50.76 | 2 |
| males | BG, BMI, CH, DBP, PP, VR | *a*(*t*)=const | <0.0001 | 34.19 | 1 |
| males | BG, BMI, CH, DBP, PP, VR | *f*_1_(*t*)=const | <0.0001 | 952.48 | 1 |
| males | BG, BMI, CH, DBP, PP, VR | *f*_1_(*t*)=0 | <0.0001 | 9222.22 | 2 |
| males | BG, BMI, CH, DBP, PP, VR | *Q*(*t*)=const | <0.0001 | 26.10 | 1 |
| males | BG, BMI, CH, DBP, PP, VR | *Q*(*t*)=0 | <0.0001 | 50.87 | 2 |
| males | BG, BMI, CH, PP, SBP, VR | *a*(*t*)=const | <0.0001 | 39.99 | 1 |
| males | BG, BMI, CH, PP, SBP, VR | *f*_1_(*t*)=const | <0.0001 | 843.82 | 1 |
| males | BG, BMI, CH, PP, SBP, VR | *f*_1_(*t*)=0 | <0.0001 | 9703.38 | 2 |
| males | BG, BMI, CH, PP, SBP, VR | *Q*(*t*)=const | <0.0001 | 24.52 | 1 |
| males | BG, BMI, CH, PP, SBP, VR | *Q*(*t*)=0 | <0.0001 | 43.06 | 2 |
| males | BG, BMI, CH, DBP, HC, VR | *a*(*t*)=const | 1 | 0.00 | 1 |
| males | BG, BMI, CH, DBP, HC, VR | *f*_1_(*t*)=const | <0.0001 | 418.08 | 1 |
| males | BG, BMI, CH, DBP, HC, VR | *f*_1_(*t*)=0 | <0.0001 | 8810.89 | 2 |
| males | BG, BMI, CH, DBP, HC, VR | *Q*(*t*)=const | <0.0001 | 19.26 | 1 |
| males | BG, BMI, CH, DBP, HC, VR | *Q*(*t*)=0 | <0.0001 | 45.97 | 2 |
| males | BG, BMI, CH, DBP, HC, SBP | *a*(*t*)=const | 0.1289 | 2.31 | 1 |
| males | BG, BMI, CH, DBP, HC, SBP | *f*_1_(*t*)=const | <0.0001 | 750.62 | 1 |
| males | BG, BMI, CH, DBP, HC, SBP | *f*_1_(*t*)=0 | <0.0001 | 8442.44 | 2 |
| males | BG, BMI, CH, DBP, HC, SBP | *Q*(*t*)=const | <0.0001 | 18.12 | 1 |
| males | BG, BMI, CH, DBP, HC, SBP | *Q*(*t*)=0 | <0.0001 | 46.44 | 2 |
| males | BG, BMI, CH, DBP, HC, PP | *a*(*t*)=const | 0.0069 | 7.30 | 1 |
| males | BG, BMI, CH, DBP, HC, PP | *f*_1_(*t*)=const | <0.0001 | 614.61 | 1 |
| males | BG, BMI, CH, DBP, HC, PP | *f*_1_(*t*)=0 | <0.0001 | 8793.94 | 2 |
| males | BG, BMI, CH, DBP, HC, PP | *Q*(*t*)=const | <0.0001 | 18.09 | 1 |
| males | BG, BMI, CH, DBP, HC, PP | *Q*(*t*)=0 | <0.0001 | 46.16 | 2 |
| males | BG, BMI, CH, HC, PP, SBP | *a*(*t*)=const | 0.0002 | 14.29 | 1 |
| males | BG, BMI, CH, HC, PP, SBP | *f*_1_(*t*)=const | <0.0001 | 518.93 | 1 |
| males | BG, BMI, CH, HC, PP, SBP | *f*_1_(*t*)=0 | <0.0001 | 9172.33 | 2 |
| males | BG, BMI, CH, HC, PP, SBP | *Q*(*t*)=const | <0.0001 | 17.20 | 1 |
| males | BG, BMI, CH, HC, PP, SBP | *Q*(*t*)=0 | <0.0001 | 40.01 | 2 |
